# On the transmit field inhomogeneity correction of relaxation‐compensated amide and NOE CEST effects at 7 T

**DOI:** 10.1002/nbm.3687

**Published:** 2017-01-23

**Authors:** Vitaliy Khlebnikov, Johannes Windschuh, Jeroen C.W. Siero, Moritz Zaiss, Peter R. Luijten, Dennis W.J. Klomp, Hans Hoogduin

**Affiliations:** ^1^ Department of RadiologyUniversity Medical Center UtrechtUtrechtThe Netherlands; ^2^ Division of Medical Physics in RadiologyDeutsches Krebsforschungszentrum (DKFZ) [German Cancer Research Center]HeidelbergGermany; ^3^ Spinoza Centre for NeuroimagingAmsterdamThe Netherlands

**Keywords:** *B*_1_ correction, Bloch‐McConnell equations, relaxation‐compensated amide‐CEST, relaxation‐compensated NOE CEST, transmit field inhomogeneity

## Abstract

High field MRI is beneficial for chemical exchange saturation transfer (CEST) in terms of high SNR, CNR, and chemical shift dispersion. These advantages may, however, be counter‐balanced by the increased transmit field inhomogeneity normally associated with high field MRI. The relatively high sensitivity of the CEST contrast to *B*_1_ inhomogeneity necessitates the development of correction methods, which is essential for the clinical translation of CEST. In this work, two *B*_1_ correction algorithms for the most studied CEST effects, amide‐CEST and nuclear Overhauser enhancement (NOE), were analyzed. Both methods rely on fitting the multi‐pool Bloch‐McConnell equations to the densely sampled CEST spectra. In the first method, the correction is achieved by using a linear *B*_1_ correction of the calculated amide and NOE CEST effects. The second method uses the Bloch‐McConnell fit parameters and the desired *B*_1_ amplitude to recalculate the CEST spectra, followed by the calculation of *B*_1_‐corrected amide and NOE CEST effects. Both algorithms were systematically studied in Bloch‐McConnell equations and in human data, and compared with the earlier proposed ideal interpolation‐based *B*_1_ correction method. In the low *B*_1_ regime of 0.15–0.50 μT (average power), a simple linear model was sufficient to mitigate *B*_1_ inhomogeneity effects on a par with the interpolation *B*_1_ correction, as demonstrated by a reduced correlation of the CEST contrast with *B*_1_ in both the simulations and the experiments.

## 1 INTRODUCTION

Chemical exchange saturation transfer (CEST) is a magnetization transfer (MT)‐based contrast allowing the low concentration metabolite pools bearing exchangeable protons to be detected indirectly through the abundant exchange‐mediating water proton pool.^1^, ^2^, ^3^, ^4^ The water signal attenuation, originating from the saturation transfer of the irradiated protons of interest by chemical exchange to the water protons, is detected via the CEST spectrum (also known as a *Z*‐spectrum).

The CEST technique in high field MRI (HF‐MRI) has generated much interest in the imaging of metabolites.^5^, ^6^, ^7^, ^8^, ^9^ Two of the most studied CEST effects are amide‐CEST^10^, ^11^, ^12^ and the relayed nuclear Overhauser enhancement (NOE).^13^, ^14^ Amide‐CEST, which is believed to originate from the cytosolic amide metabolites, has found its application in glioma grading,^15^, ^16^, ^17^ cancer therapy monitoring,^18^, ^19^ and differentiation between necrosis and tumor regrowth.^20^ NOE originates from aliphatic and olefinic protons of the cellular mobile macromolecule effect and has been reported to be linked to tissue cellularity^21^ and cellular membrane fluidity.^22^

The CEST contrast is unique in providing quantitative metabolite‐specific information. To accurately resolve physiological spatial variations in the CEST contrast it is crucial to minimize contrast variations due to system imperfections. While CEST at HF‐MRI benefits from high SNR, CNR, and chemical shift dispersion, it suffers from the consequent increased transmit field inhomogeneity. The relatively high sensitivity of the CEST contrast to *B*_1_ inhomogeneity necessitates the development of correction methods, which is essential for the clinical translation of CEST. Previously, Windschuh et al. proposed an interpolation‐based approach to correct *Z*‐spectra and CEST contrast for *B*_1_ inhomogeneity.^23^ In this approach, the densely sampled *Z*‐spectra are acquired at at least two different *B*_1_ levels, and *B*_1_ correction of *Z*‐spectra and isolated CEST contrast is achieved by spline interpolation of the multiple *B*_1_ data to a *B*_1_ of interest. However, this approach may not be possible in a clinical setting, where the scan time is very limited.

In this work, two methods that require only one CEST dataset at a particular *B*_1_ level and a relative *B*_1_ map as a reference are compared. Both methods rely on fitting the multi‐pool Bloch‐McConnell equations^24^ to the densely sampled *Z*‐spectra using a *B*_1_ map as a reference. In the first method, an assumption is made about a linear relationship of CEST effects with *B*_1_. The *B*_1_ correction is achieved by using a linear *B*_1_ correction of the calculated amide and NOE CEST effects. The second method is based on an assumption that the Bloch‐McConnell estimated fit parameters other than *B*_1_ are independent of the actual *B*_1_. The estimated fit parameters and the desired *B*_1_ amplitude are used to recalculate the *Z*‐spectra followed by the calculation of *B*_1_‐corrected amide and NOE CEST effects. Both approaches were first evaluated in simulated data and subsequently tested in data from healthy human brain.

## 2 METHODS

### 2.1 Generation of simulated CEST spectra

Four‐pool (water, amide‐CEST, NOE and MT) Bloch‐McConnell equations were solved numerically^25^ assuming the following white matter (WM) pool parameters^26^: (i) water (*T*_1_/*T*_2_ = 1.2 s/40 ms); (ii) amide‐CEST (*T*_1_/*T*_2_ = 1 s/10 ms, exchange rate 50 Hz, pool size ratio 0.13%, chemical shift 3.5 ppm); (iii) NOE (*T*_1_/*T*_2_ = 1 s/0.3 ms, pool size ratio 6%, exchange rate 10 Hz, chemical shift −3.5 ppm); and (iv) MT (*T*_1_/*T*_2_ = 1 s/10 μs, pool size ratio 11%, exchange rate 50 Hz, chemical shift −2.4 ppm). Even though the NOE effect (−3.5 ppm) was shown to be composed of multiple fine structures,^13^ we chose to approximate it with the single offset due to the use of short pulses with high bandwidth in this work. Due to large insensitivity of simulations to *T*_1_ values of other than water pools, the *T*_1_ of amide‐CEST, NOE and MT was fixed to 1 s, as suggested previously.^27^ The sequence parameters used in the simulations are the same as in the data acquisition (see later), except for the *B*_1_ level extended up to 1.8 μT (average power). The simulations were based on the assumption that there are only four pools in the system and that the only interactions are with water.

### 2.2 Data acquisition

In this report, we made a retrospective analysis of the data in Reference 23. *In vivo* experiments were performed on a 7 T MR whole‐body system (Magnetom; Siemens, Erlangen, Germany) using a Tx/Rx head coil (Tx, one channel; Rx, 24 channels). The CEST protocol was as follows^28^: saturation consisted of a train of 120, 15 ms Gaussian pulses interleaved with a GR‐spoiler, duty cycle 60%; for readout a single‐shot 2D gradient echo sequence (GRE) was used with GRAPPA acceleration factor 2, *T*_R_/*T*_E_/FA = 7.4 ms/3.6 ms/10°, matrix 128 × 128, slice thickness 5 mm. Total scan time was 4 min 7 s. *Z*‐spectra were sampled at 66 frequency offsets distributed unevenly between ±500 ppm (500 ppm offset was used for normalization). The CEST sequence was performed at eight different *B*_1_ levels: 0.14, 0.29, 0.43, 0.50, 0.58, 0.65, 0.72, and 0.80 μT. *B*_1_ level refers to the nominally set, average power of the saturation pulse throughout the paper. *B*_0_ inhomogeneity was corrected using the WASSR method.^29^ A 2D flip‐angle map was based on a single‐shot GRE sequence: a rectangular preparation pulse (2 ms) with nominal flip angle 90°, *T*_E_/*T*_R_ = 2.42 ms/5000 ms. The transmitter voltage and thus the nominal *B*_1_ values were calibrated on the basis of this flip angle map. A relative map of irradiation amplitude (r*B*_1_(*x*, *y*)) was produced by the normalization of this flip‐angle map by the nominal flip angle. The actual irradiation amplitude *B*_1_ in each pixel (*x*, *y*) was assigned employing the relative *B*_1_ map r*B*_1_(*x*, *y*) by *B*1(x, *y*) = r*B*1(*x*, *y*)*B*1 , nom, where *B*_1,nom_ is the nominal *B*_1_ value as chosen in the protocol settings. A *T*_1_‐weighted anatomical image was used to produce white matter (WM) and grey matter (GM) masks in FSL (FMRIB v6.0, UK).

### 2.3 Fitting Bloch‐McConnell equations to the data

The four (water, amide‐CEST, NOE and MT) and six (water, amide‐CEST, NOE, MT, amine‐CEST^23^ and NOE*^22^) pool Bloch‐McConnell equations were used to fit the simulated and the *in vivo Z*‐spectra, respectively. The data was fitted at a single *B*_1_ level at any given time. Since the saturation duration in the employed sequence is less than water *T*_1_, the saturation duration was taken into account in data fitting.^25^ The choice of six pools to fit the *in vivo Z*‐spectra was based on the results of fitting a few test *Z*‐spectra by incrementing the number of pools and monitoring the goodness‐of‐fit statistics. Increasing the number of pools from four to six reduced the sum of squared errors by 50% (*F*‐test, *p* < 0.01). The fitting was done employing a non‐linear least squares constrained optimization algorithm (lsqcurvefit function in MATLAB) and using the pool parameters^26^, ^30^, ^31^, ^32^, ^33^ in Table 1. The goodness of fit was examined using Curve Fitting Toolbox™ in MATLAB with the following metrics: (i) the sum of squared errors; (ii) *R*‐square; (iii) adjusted *R*‐square; and (iv) root mean squared error.

**Table 1:** The parameters used for fitting the Bloch‐McConnell equations to CEST spectra

		Water	Amide‐CEST	NOE (Pool 1)	MT	Amine‐CEST	NOE* (Pool 2)
*T*_1_ (s)	*X*_0_	1.5	1	1	1	1	1
	LB	1.0	—	—	—	—	—
	UB	2.5	—	—	—	—	—
*T*_2_	*X*_0_	50 ms	10 ms	0.5 ms	20 μs	10 ms	0.5 ms
	LB	20 ms	0.2 ms	0.1 ms	10 μs	0.2 ms	0.1 ms
	UB	70 ms	15 ms	10 ms	80 μs	15 ms	10 ms
Δ*ω* (ppm)	*X*_0_	0	3.5	−3.5	−2.4	2.0	−1.6
	LB	−0.1	3.0	−4.0	−4.0	1.5	−1.8
	UB	0.1	4.0	−3.0	−2.0	2.5	−1.4
*M*_0_ (%)	*X*_0_	—	0.1	4.5	9	0.01	1
	LB	—	0	0	0	0	0
	UB	—	0.2	13.5	27	0.10	10
*k* (Hz)	*X*_0_	—	50	10	50	1 000	10
	LB	—	0	0	0	0	0
	UB	—	600	50	150	10 000	50

*X*_0_, LB and UB represent the initial guess and lower and upper bounds, respectively.

The only parameters fixed in the fit were the actual *B*_1_ (Equation (1)) and *T*_1_ (set to unity) for all pools except water.

(1) $$B_{1}\left(\text{actual},\mathrm{\mu T}\right)=B_{1}\left(\text{nominal},\mathrm{\mu T}\right)B_{1}\left(\text{relative}\right).$$

To correct for the effects of the traditional MT and direct water saturation, the amide‐CEST effect size (contribution to the *Z*‐spectrum) was quantified by the pool difference method using the inverse metrics^34^, ^35^:

(2) $${\text{MTR}}_{\text{Rex,amide}},=\frac{1}{M_{z}\left(3.5\mathrm{ppm},M_{b}=1\right)/M_{0}}-\frac{1}{M_{z}\left(3.5\mathrm{ppm},M_{b}=0\right)/M_{0}}$$

where MTR_Rex,amide_ is the effect size of the cytosolic amides, *M*_*z*_(Δ*ω*, *M*_b_) is the signal in the *Z*‐spectrum at Δ*ω* (Δ*ω* = 3.5 ppm for amide‐CEST), *M*_0_ is the equilibrium magnetization at the normalization offset Δ*ω =* 500 ppm and *M*_b_ is the amplitude of the amide compartment (*M*_b_ = 0 and *M*_b_ = 1 for the system without and with amide‐CEST pool, respectively). (*M*_b_ = 0) − (*M*_b_ = 1) gives the amide‐CEST pool, hence the name “pool difference method”. The pool difference method used in this work was based on the inverse metrics approach and hence the reciprocals in Equation (2).

A similar equation applies to the NOE pool (MTR_Rex,NOE_) at Δ*ω* = −3.5 ppm. The apparent exchange dependent relaxation (AREX or relaxation compensated MTR_Rex_)^34^, ^35^, ^36^, ^37^ was not calculated, since the *B*_1_ dependence remains the same for MTR_Rex_ and AREX. The strength of a linear relationship between paired data was determined by the Pearson correlation coefficient (*R*).

### 2.4 Bloch‐McConnell equation *B*_1_ correction

**Figure 1:**
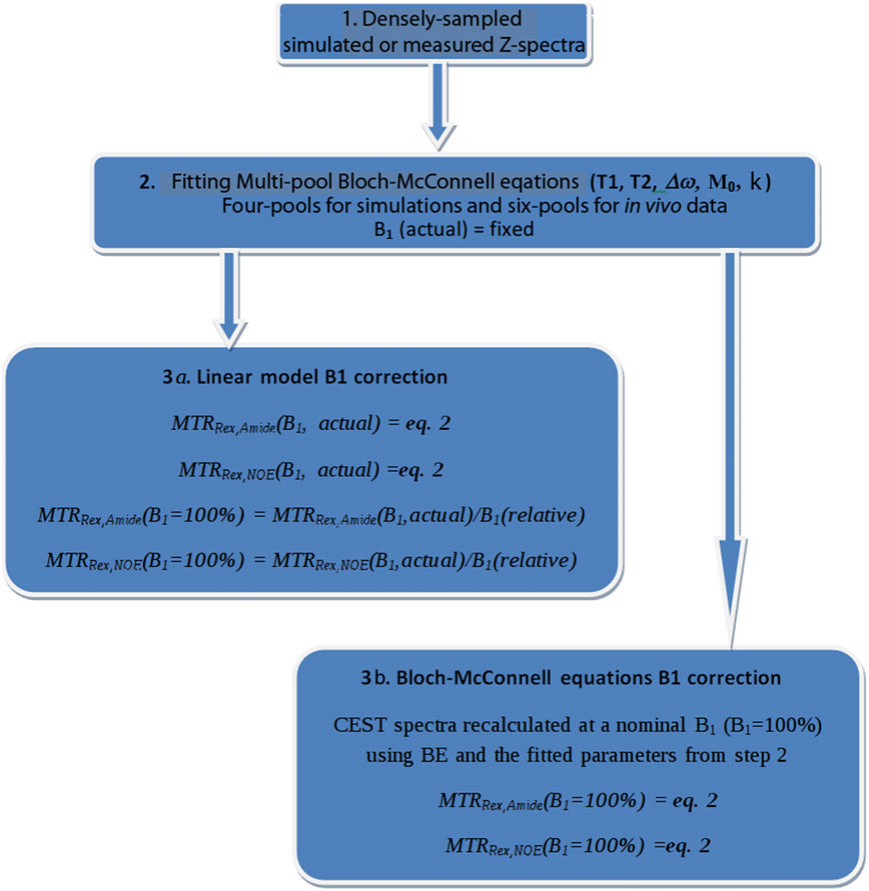
A flowchart representing the steps for implementing a linear model and BE *B*_1_ correction algorithms.

The workflow of the Bloch‐McConnell equation (BE) *B*_1_ correction algorithm is illustrated in the flowchart (Figure 1). First, the densely sampled *Z*‐spectra are acquired. Second, multi‐pool BEs are fitted to the spectra to determine *T*_1_ (spin–lattice relaxation time), *T*_2_ (spin–spin relaxation time), Δ*ω* (chemical shift with respect to water), *M*_0_ (pool size), and *R*_ex_ (exchange rate). The only parameters fixed during the fitting process are *B*_1_(actual), since this parameter is known from a *B*_1_ map used as a reference (Equation (1)), and *T*_1_ (fixed to unity) for all pools other than water. Then, the *Z*‐spectra are recalculated at a nominal *B*_1_ level (*B*_1_ = 100%) using the previously fitted BE parameters. Finally, the *B*_1_‐corrected effect size of amide and NOE is isolated using Equation (2).

### 2.5 Linear model *B*_1_ correction

The first and the second steps of the linear *B*_1_ correction algorithm are identical to those of the BE *B*_1_ correction algorithm (Figure 1). In the third step, the effect size of amide and NOE is isolated using Equation (2) and a linear *B*_1_ correction is achieved by division of the isolated effects by the relative *B*_1_.

### 2.6 Comparison with interpolation‐based *B*_1_ correction

Both *B*_1_ correction algorithms analyzed in this work were compared with the ideal interpolation‐based *B*_1_ correction approach.^23^

The contrast maps of amide and NOE were generated at all *B*_1_ levels as described in the flowchart (Figure 1, Steps 1 and 2) and using Equation (2) to extract the effect sizes. The *B*_1_‐corrected maps of both amide and NOE effects were produced by voxel‐wise spline interpolation of the corresponding MTR_Rex_ maps at all *B*_1_ levels to a *B*_1_ of 0.43 μT using the eight‐point contrast *B*_1_ correction as explained in Reference 23.

## 3 RESULTS

### 3.1 Numerical simulations

**Figure 2:**
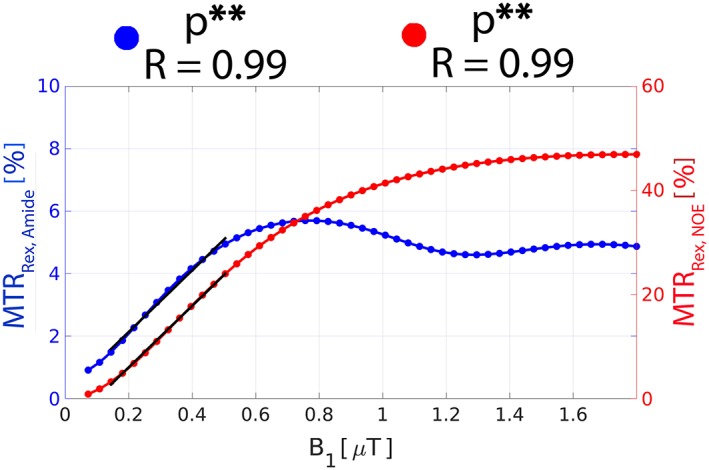
The Bloch‐McConnell equation simulated *B*_1_ dependence of the effect size of MTR_Rex,amide_ and MTR_Rex,NOE_ in WM. The straight black lines represent the linear regression relation between the corresponding metrics and *B*_1_ in the *B*_1_ range 0.1–0.5 μT. The Pearson correlation coefficient (*R*) and the corresponding *p*‐value are provided. **Statistical significance at the level *p* < 0.005.

In Figure 2, the BEs were used to simulate the *B*_1_ dependence of amide‐CEST (MTR_Rex,amide_) and NOE (MTR_Rex,NOE_) effect size. In the low *B*_1_ regime (0.1–0.5 μT), the *B*_1_ dependence of the effects is largely linear: *R* = 0.99 (*p* < 0.005) for both MTR_Rex,amide_ and MTR_Rex,NOE._ There are noticeable rotation effects for MTR_Rex,amide_ at a *B*_1_ above 0.8 μT.^38^, ^39^, ^40^

**Figure 3:**
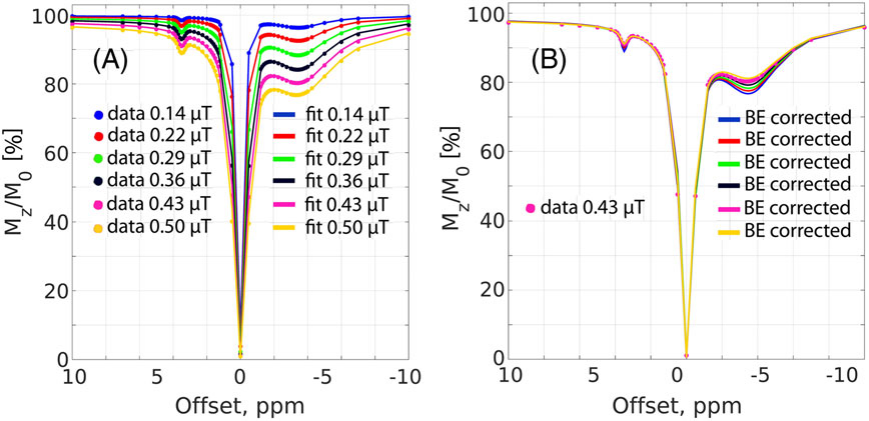
A, The four‐pool Bloch‐McConnell equation simulated spectra (colored markers) at various *B*_1_ levels and their corresponding four‐pool Bloch‐McConnell fits (colored solid lines). B, Same as A for the colored markers, but the colored solid lines represent BE‐corrected spectra recalculated at a *B*_1_ of 0.43 μT (assumed to be nominal *B*_1_ level) using the corresponding fitting parameters from A. A Gaussian noise of 1% (of the signal at 500 ppm) was added to the simulated data.

**Figure 4:**
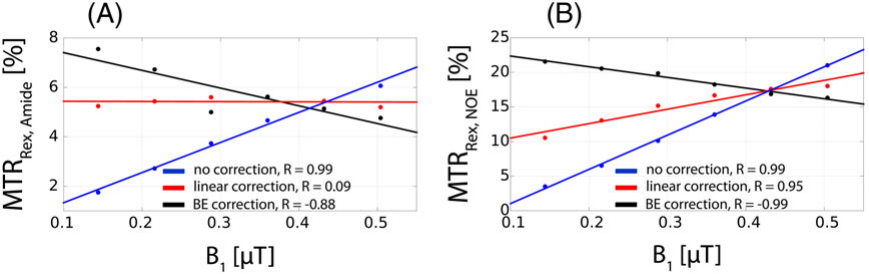
A, The comparison of uncorrected (isolated from Figure 3A), the linear model *B*_1_‐corrected (isolated from Figure 3A with the subsequent linear *B*_1_ correction), and the BE *B*_1_‐corrected (isolated from Figure 3B) MTR_Rex,amide_ effect size as a function of *B*_1_. B, The same as A but for MTR_Rex,NOE_ effect size. For the linear *B*_1_ correction, a *B*_1_ of 0.43 μT was assumed to be nominal *B*_1_ (100%). All other *B*_1_ levels were translated to percentages accordingly. The straight colored lines represent the linear regression relation between the corresponding metrics and *B*_1_. The Pearson correlation coefficient (*R*) is shown in each subfigure.

The concept of the BE *B*_1_ correction is shown in Figure 3, where a series of *Z*‐spectra was simulated in the *B*_1_ range 0.1–0.5 μT and subsequently fitted using BEs (Figure 3A). The BE fit parameters from Figure 3A were used to recalculate all of the spectra at a *B*_1_ of 0.43 μT (Figure 3B). The overlap of the BE *B*_1_‐corrected *Z*‐spectra suggest that BEs may correct the effects of *B*_1_ inhomogeneity. Assuming a nominal *B*_1_ of 0.43 μT, the *B*_1_ range 0.1–0.5 μT used in the simulations (Figure 3A) is expected in the *in vivo* experiments because of *B*_1_ inhomogeneity (typically 60–120%). The effects of amide‐CEST (MTR_Rex,amide_) and NOE (MTR_Rex,NOE_) isolated from these *Z*‐spectra are termed uncorrected, i.e. without correction for the *B*_1_ inhomogeneity. In Figure 4, the uncorrected effect size of MTR_Rex,amide_ (Figure 4A, blue) and MTR_Rex,NOE_ (Figure 4B, blue) is plotted versus *B*_1_, and compared with those yielded by the linear (red) and the BE *B*_1_ correction (black) algorithms. As expected, both MTR_Rex,amide_ and MTR_Rex,NOE_ uncorrected effects have a strong positive *B*_1_ correlation (*R* = 0.99 for both effects). The BE *B*_1_ correction over‐ and underestimated MTR_Rex,amide_ effect size at low and high *B*_1_, respectively (*R* = −0.88, black), whereas the linear *B*_1_ correction showed a relatively stable effect size across the whole *B*_1_ range simulated (*R* = 0.09, red). For MTR_Rex,NOE_, the BE *B*_1_ correction also over‐ and underestimated the effect size at low and high *B*_1_, respectively (*R* = 0.99, black), whereas the linear *B*_1_ correction reduced the effect of *B*_1_ inhomogeneity (*R* = 0.95, red). In addition, the BE *B*_1_ correction reversed the sign of the Pearson correlation coefficient due to the over‐ and under‐compensation at low and high *B*_1_, respectively.

### 3.2 Experimental results

**Figure 5:**
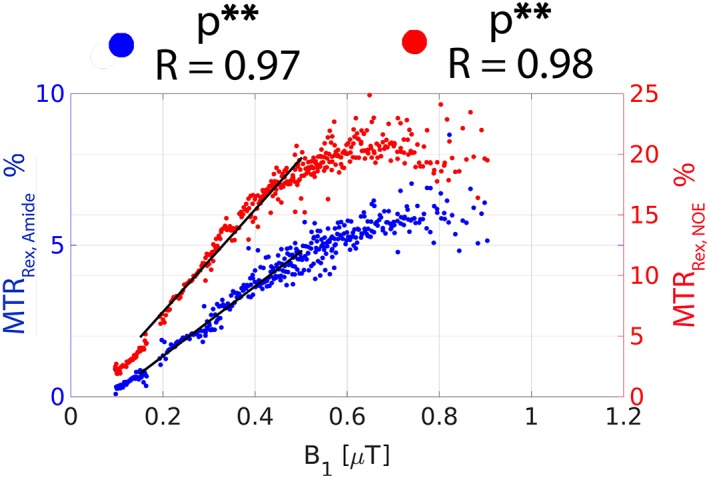
The experimentally derived plots of MTR_Rex,amide_ and MTR_Rex,NOE_ as a function of the actual *B*_1_ values in WM. The traces were obtained by segmenting the relative *B*_1_ map (Figure 7A) into the different regions between 50% and 150% in steps of 1% and calculating the corresponding MTR_Rex,amide_ and MTR_Rex,NOE_ contrast resulting from all available CEST datasets. The straight black lines represent the linear regression relation between the corresponding metrics and *B*_1_ in the *B*_1_ range 0.1–0.5 μT. The Pearson correlation coefficient (*R*) and the corresponding *p*‐value are provided. **Statistical significance at the level *p* < 0.005.

**Figure 6:**
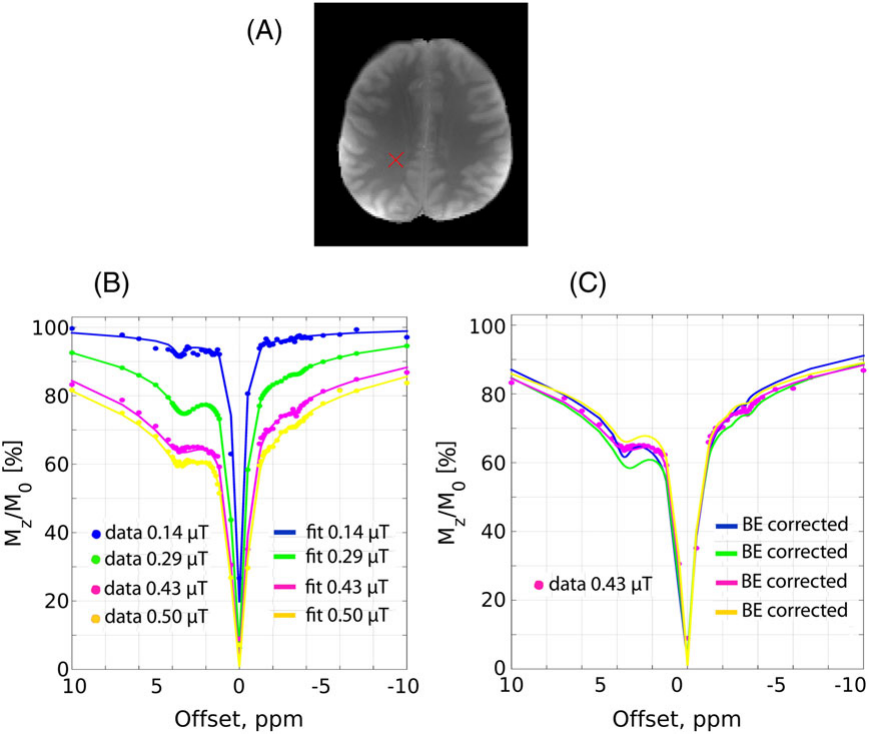
A, A CEST image of the healthy human brain with the cross marking the origin of the CEST spectra shown in B and C. B, The *in vivo* CEST spectra (colored markers) at various *B*_1_ levels and their corresponding six‐pool Bloch‐McConnell fits (colored solid lines). C, Same as B for the colored markers, but the colored solid lines represent BE‐corrected spectra recalculated at a *B*_1_ of 0.43 μT (assumed to be nominal *B*_1_ level) using the corresponding fitting parameters from B.

The experimentally derived *B*_1_ dependence of MTR_Rex,amide_ and MTR_Rex,NOE_ is plotted in Figure 5. As predicted in the simulations (Figure 2), both effects are linear with *B*_1_ in the range 0.1–0.5 μT (*R* = 0.97 and *R* = 0.98 for MTR_Rex,amide_ and MTR_Rex,NOE_, respectively), after which the effects start to level off. A nominal *B*_1_ level of 0.43 μT was chosen to compare the performance of the linear model and the BE *B*_1_ correction algorithms, because it is still in the linear *B*_1_ regime and yields a good effect size of both of MTR_Rex,amide_ and MTR_Rex,NOE_. At this power level, the effect size of amide and NOE is reduced by 15% and 10%, respectively, relative to their corresponding maxima. In Figure 6, the correction of transmit field inhomogeneity effects by BEs is demonstrated using the experimental *in vivo* data. The CEST spectra from white matter (Figure 6A) at four power levels, 0.14 μT, 0.29 μT, 0.43 μT, and 0.50 μT, were fitted with BEs (Figure 6B) and subsequently recalculated at a *B*_1_ of 0.43 μT (Figure 6C), resulting in the overlap of BE *B*_1_‐corrected spectra.

**Figure 7:**
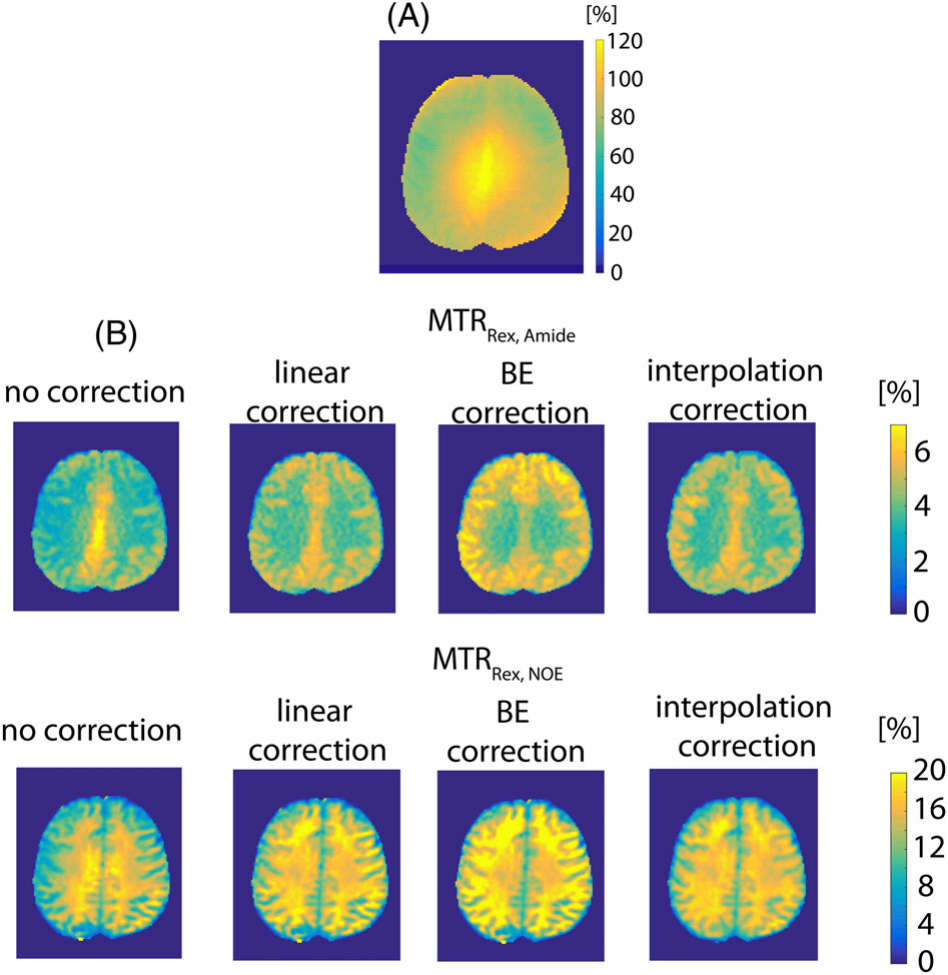
A, A relative *B*_1_ map. B, Comparison of the experimentally derived uncorrected, the linear model *B*_1_‐corrected, the BE *B*_1_‐corrected and the interpolation‐based *B*_1_‐corrected contrast for MTR_Rex,amide_ (top row) and MTR_Rex,NOE_ (bottom row).

As expected from the simulations (Figure 2) and the experiments (Figure 5), the visual inspection reveals a strong correlation of uncorrected maps of MTR_Rex,amide_ and MTR_Rex,NOE_ (Figure 7B) with the relative *B*_1_ map (Figure 7A): a high signal in the center and low at the sides. The linear *B*_1_ correction appears to alleviate the issue of *B*_1_ inhomogeneity effectively and create a homogeneous contrast for both MTR_Rex,amide_ and MTR_Rex,NOE_, whereas the BE *B*_1_ correction results in the over‐ and under‐correction of *B*_1_ inhomogeneity effects at low and high power, respectively. Interestingly, both the linear model and the interpolation produced contrast maps of similar quality for both MTR_Rex,amide_ and MTR_Rex,NOE_.

**Figure 8:**
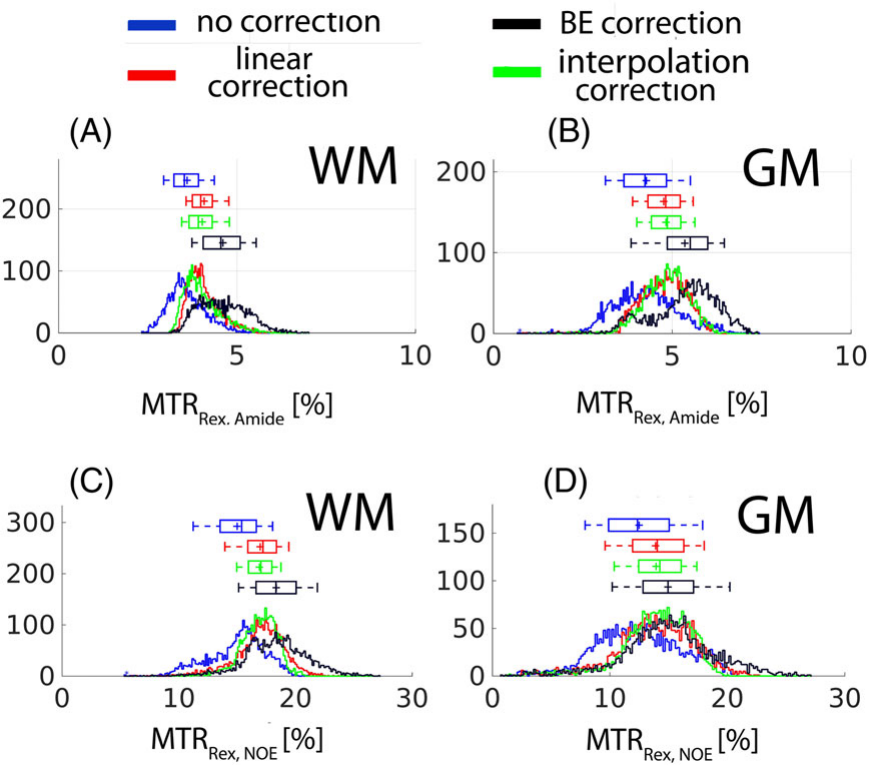
The histograms of the images shown in Figure 7B. A,B, WM and GM, respectively, for MTR_Rex,amide_. C,D, WM and GM, respectively, for MTR_Rex,NOE_. The box and whiskers above each histogram contain values of 25–75% and 9–91%, respectively.

A graphical representation of the contrast distribution is another way to compare the performance of the linear model and the BE *B*_1_‐correction approaches. When compared with the uncorrected contrast, the linear *B*_1_ correction effectively reduced the data dispersion (reflected in the box and whiskers above each distribution) for both MTR_Rex,amide_ (Figure 8A,B) and MTR_Rex,NOE_ (Figure 8C,D). Both the linear and the interpolation *B*_1_ corrections seem to produce similar contrast distributions. The BE *B*_1_ correction algorithm clearly over‐corrected data, resulting in a very broad distribution, which can also be seen by visual inspection of the images in Figure 7B.

**Figure 9:**
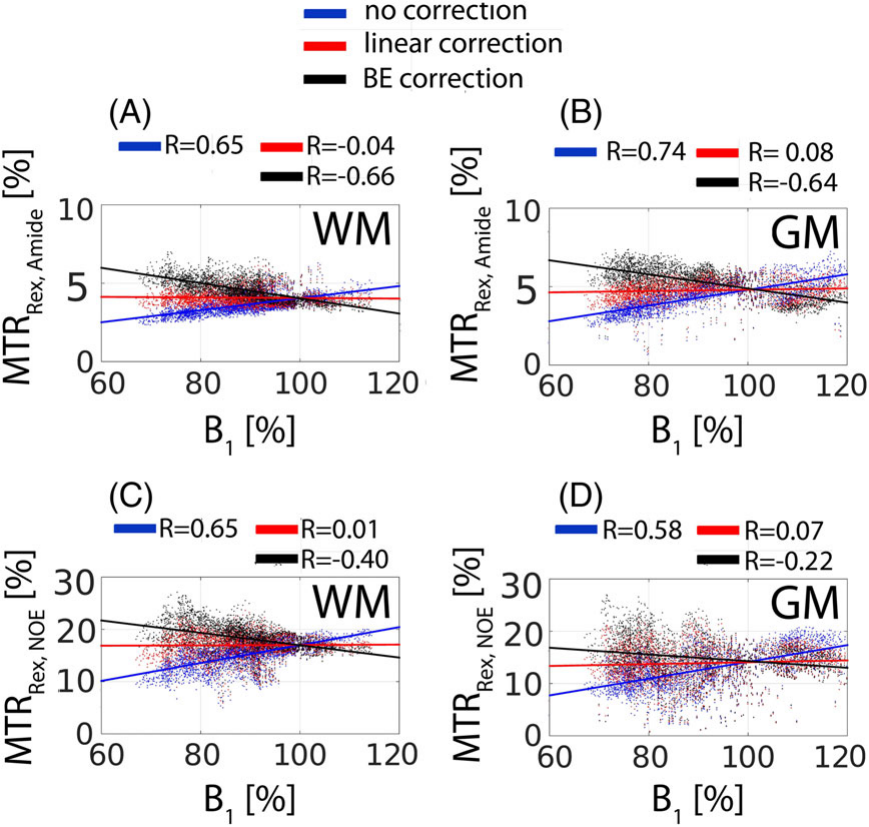
The voxel‐wise correlation of the image contrast (Figure 7B) with the relative *B*_1_ map (Figure 7A). A,B, WM and GM, respectively, for MTR_Rex,amide_. C,D, WM and GM, respectively, for MTR_Rex,NOE_. The linear colored lines represent the linear regression. The Pearson correlation coefficient (*R*) is shown in each subfigure.

As expected, a strong correlation of the uncorrected contrast MTR_Rex,amide_ (Figure 9A,B) and MTR_Rex,NOE_ (Figure 9C,D) with *B*_1_ is evident in Figure 9. For example, the correlation coefficient (*R*) of the uncorrected MTR_Rex,amide_ (Figure 9A) and MTR_Rex,NOE_ (Figure 9C) was found to be 0.65 in WM. The linear *B*_1_ model virtually nullified the correlation by reducing the correlation coefficients to −0.04 (Figure 9A) and 0.01 (Figure 9C) for MTR_Rex,amide_ and MTR_Rex,NOE_, respectively. In line with the simulations in Figure 4 and the experimental results shown in Figure 7B, Figure 8 and Figure 9, the BE *B*_1_ correction algorithm resulted in over‐ and under‐correction at low and high *B*_1_, respectively.

## 4 DISCUSSION

In this work, we compared two algorithms for *B*_1_ correction of amide‐CEST (MTR_Rex,amide_) and NOE (MTR_Rex,NOE_) effects at 7 T. Both methods rely on fitting the multi‐pool BEs to densely sampled CEST spectra to extract the effects. The first algorithm is based on a simple linear model *B*_1_ correction of the isolated effects. The second algorithm uses the fit parameters to recalculate the *Z*‐spectra at a *B*_1_ of interest followed by extraction of the *B*_1_‐corrected effects. Both algorithms were compared in the BE simulated and experimental *in vivo* data of the brain of a healthy volunteer. In the BE simulated data, a simple linear model appeared to be more effective in mitigating *B*_1_ inhomogeneity effects. In line with the simulations, the linear *B*_1_ correction outperformed the BE *B*_1_ correction algorithm in the experimental data obtained in the healthy human brain. The linear *B*_1_ correction generated homogeneous image contrast for both MTR_Rex,amide_ and MTR_Rex,NOE_ and resulted in almost zero correlation of the effects with *B*_1_, whereas the BE *B*_1_ correction algorithm greatly overcompensated the areas with low *B*_1_, thereby increasing the contrast dispersion.

### 4.1 Numerical simulations

The four‐pool BE simulations suggest a linear *B*_1_ dependence of MTR_Rex,amide_ (*R* = 0.99, *p* < 0.005) and MTR_Rex,NOE_ (*R* = 0.99, *p* < 0.005) in the low *B*_1_ range 0.1–0.5 μT (Figure 2). This opens up the possibility of a simple linear correction of *B*_1_ inhomogeneity of both effects in this low *B*_1_ regime. The MTR_Rex,amide_ rotation effects^38^, ^39^, ^40^ may pose a challenge when making *B*_1_ corrections at higher *B*_1_ levels.

The perfectly fitted simulated spectra (Figure 3A) and the visual inspection of the overlapping BE *B*_1_‐corrected CEST spectra (Figure 3B) suggest that fixing only one *B*_1_ parameter in the BEs and allowing the rest to vary within reasonable constraints is sufficient to fit and correct the CEST spectra for *B*_1_ inhomogeneity in this low *B*_1_ range, 0.1–0.5 μT. However, care must be taken since the similarity between the BE *B*_1_‐corrected spectra does not guarantee that they contain similar CEST features, i.e. MTR_Rex,amide_ and MTR_Rex,NOE_, which are of interest and should be isolated from the spectra. Therefore, for the fair comparison of both *B*_1_ correction algorithms, we chose to compare them in terms of the *B*_1_‐corrected MTR_Rex,amide_ and MTR_Rex,NOE_ effects.

The BEs incorporate the effect of chemical exchange and are known to describe the exchange‐mediated processes precisely.^24^ However, many parameters are correlated, e.g. *T*_2_ and *k*, *k* and *M*_0_,^41^ which makes it extremely difficult to determine those unambiguously from fitting a single CEST spectrum at one power level. This uncertainty propagates further when recalculating the spectra at a nominal *B*_1_ (*B*_1_ = 100%) and extracting the *B*_1_‐corrected contrast, since *R*_ex_ has an effect on the contrast *B*_1_ sensitivity.^42^ The over‐ and under‐correction of MTR_Rex,amide_ and MTR_Rex,NOE‐_contrast by the BE *B*_1_ correction algorithm at low and high *B*_1_ levels, respectively (Figure 4A,B, respectively), can be attributed to the parameter correlation. The better performance of the linear *B*_1_ correction can be explained by the absence of an extra step involving the Bloch equation numerical calculations. In addition, a linear relation of MTR_Rex,amide_ and MTR_Rex,NOE_ with *B*_1_ was predicted in the simulations (Figure 2).

The simulation results should be treated with caution, since an assumption was made on the initial pool parameters. In addition, there is no agreement in the literature on the number of pools and the simulation parameters.^26^, ^30^, ^31^, ^32^, ^33^

### 4.2 Experimental results

Fitting *in vivo* CEST spectra using BEs is a challenge since the exact number of pools is unknown beforehand. In this work, we chose to use six‐pool BEs to approximate the *in vivo* complexity. The strong positive linear relationships of MTR_Rex,amide_ (*R* = 0.97, *p* < 0.005) and MTR_Rex,NOE_ (*R* = 0.98, *p* < 0.005) with *B*_1_ in the range 0.1–0.5 μT (Figure 5) lead us to hypothesize that a simple linear *B*_1_ correction may be sufficient *in vivo* in this *B*_1_ range. The fact that both the simulations (Figure 2) and the experiments (Figure 5) showed a linear relation of MTR_Rex,amide_ and MTR_Rex,NOE_ with *B*_1_ in the low *B*_1_ regime (0.1–0.5 μT) suggests that the simulation parameters chosen in this study were within the acceptable range. The linear correction *in vivo* in this linear *B*_1_ regime comes at the cost of a small reduction in the extracted effect size compared with that obtained at a *B*_1_ level beyond the linear regime. The data dispersion in contrast of MTR_Rex,amide_ and MTR_Rex,NOE_ at high *B*_1_ in Figure 5 may be associated with the presence of labile protons with a range of exchange rates as would be expected *in vivo* as opposed to the constants used in the simulated data. The higher *B*_1_ increases sequence sensitivity to the metabolites bearing labile protons with faster exchange rates. In addition, the results were averaged across small ROIs, which may have different pool parameters. Six pools were sufficient to fit the BEs to the WM (Figure 6A) *in vivo* CEST spectra (Figure 6B) in the low *B*_1_ regime and the overlap of the BE *B*_1_‐corrected (recalculated to a nominal *B*_1_ of 0.43 μT) CEST spectra (Figure 6C) suggest that multi‐pool BEs may alleviate the issue of transmit field inhomogeneity.

The interpolation *B*_1_ correction method^23^ can be considered an ideal *B*_1_ correction approach due to its applicability to any *in vivo* system at any *B*_1_ level. Therefore, all contrast maps generated, uncorrected, linearly and BE *B*_1_ corrected, were compared with those produced by the interpolation (Figure 7B). Only the linearly *B*_1_‐corrected maps of both MTR_Rex,amide_ and MTR_Rex,NOE_ effects resemble those generated by the interpolation in terms of the image quality and the effect size, which further validates our assumption of a linear *B*_1_ correction in the low *B*_1_ regime (Figure 8 and Figure 9). For more detailed analysis of the interpolation *B*_1_ correction approach, e.g. number of *B*_1_ levels, image quality, etc., the interested reader is referred to the original work by Windschuh et al.^23^ However, the interpolation method always requires multiple acquisitions with varying *B*_1_ levels; a more elegant approach using only a single acquisition would of course be favorable. The authors also compared the interpolation *B*_1_ correction with the linear correction. For the comparison, the authors assumed a linear dependence of CEST effects up to a *B*_1_ of 0.65 μT. However, a linear model is no longer valid at this high power (Figure 5), and so the authors concluded that at least two *B*_1_ levels were necessary for *B*_1_ correction. Yet, we show that a small compromise in the effect size of amide (15%) and NOE (10%), caused by reduced *B*_1_ level to be in the linear regime, leads to a simple *B*_1_ correction method.

The data dispersion and the contrast over‐ and under‐correction by the BE *B*_1_‐correction algorithm are clearly noticeable when comparing the interpolation and the BE *B*_1_‐corrected maps (Figure 7B), the histogram (Figure 8), and the linear regression analysis (Figure 9). We attribute this to the correlation of the fitted parameters. A total of 22 parameters were fitted to the *in vivo* data and many of the parameters are highly correlated, i.e. have the same effect on CEST spectra appearance. This great number of degrees of freedom, along with the fact that many of the Bloch‐McConnell estimated fit parameters are not independent of the actual *B*_1_, may cause an unpredictable system behavior when recalculating CEST spectra at a *B*_1_ of 100% using the non‐linear system of BEs to describe a simple linear relationship. The following fit parameters were found to have a significant correlation (*R*) with *B*_1_: water *T*_1_ (−0.20), water *T*_2_ (0.29), amide *T*_2_ (−0.35), NOE *k* (−0.22), MT *k* (−0.37), MT *T*_2_ (−0.31), amine *k* (−0.19), and NOE* *k* (−0.20). While the performance of the algorithm may be further improved by measuring and fixing other parameters, e.g. exchange rate and *T*_2_, this would make this method highly inefficient since the clinical scan time is very limited. Fixing water *T*_1_, however, did not improve the performance of the BE *B*_1_‐correction algorithm. In this manuscript, the strong linear correlation between *M*_0_ (concentration) and *k* (exchange rate), which is difficult to decouple,^41^ has been exploited to our advantage. For the same effect size (amide or NOE) a low fitted *M*_0_ will be compensated by a high fitted *k* and vice versa. The *B*_1_ correction algorithms in this work apply to the effect size (a product of *M*_0_ and *k*), and so the individual parameters are less relevant as long as the BEs fit the original data.

Despite the fact that the linear *B*_1_ correction algorithm was shown only on healthy brain, it is expected to be applicable to pathological tissue as well. Abnormally high water *T*_1_ expected in tumors will scale the CEST effect,^34^, ^35^ but the linear *B*_1_ dependence of amide‐CEST and NOE effects at low power levels will not change with water *T*_1_ (Supporting Information SI1, Figure S1). The same is true for different CEST saturation parameters, e.g. saturation duration and duty cycle, as long as the average power, which takes account of the CEST saturation parameters,^43^ is low (0.1–0.5 μT). A change in water *T*_1_ and CEST saturation parameters may, however, cause a variation in amide‐CEST and NOE signal losses using the linear assumption when compared with measuring the effects at optimal *B*_1_ levels.

In this work, we opted for the use of the multi‐pool BE to extract amide and NOE features from CEST data as it is the only approach that intrinsically incorporates the sequence parameters, e.g. *B*_1_ and other CEST saturation prepulse parameters, and the physiological parameters, e.g. metabolite concentration and pH‐dependent exchange rate of labile protons with water. Yet, we expect the linear *B*_1_ correction to be applicable to the other methods used for amide and NOE isolation such as the three point method^44^, the Lorentzian difference method,^13^, ^45^ and multiple Lorentzian fitting^23^, ^46^.

## 5 LIMITATIONS

The *B*_1_ correction algorithms analyzed in this work are based on the multi‐pool Bloch‐McConnell equation fitting of densely sampled CEST spectra. An assumption is made as to the number of pools in the system, which is unknown *a priori*. This may require a test fit of the Bloch‐McConnell equations to a sample spectrum with an increasing number of pools. To determine the minimum number of pools necessary to describe the *in vivo* system of interest, the fit precision should be monitored by checking the sum of the squares of the residuals or any other appropriate measure.

## 6 CONCLUSIONS

In this work, we compared two approaches to the transmit field inhomogeneity correction of the relaxation compensated amide‐CEST and NOE effects. Both methods were compared in simulated and *in vivo* brain data obtained in a healthy human volunteer. A simple linear model for *B*_1_ correction outperformed a *B*_1_ correction algorithm based on the Bloch‐McConnell equations at the low power levels (0.1–0.5 μT). This was demonstrated by the improved image quality, reduced data dispersion and virtually nullified correlation of the CEST contrast with *B*_1_.

## Supporting information




**Sup. Figure S1**. The four‐pool Bloch‐McConnell equation simulated B_1_ dependence of the effect size of (**a**) MTR_Rex,Amide_ and (**b**) MTR_Rex,NOE_ at various water T_1_ relaxation times.
**Sup. Figure S2.** The experimentally derived plots of the three‐point method quantified MTR_Rex,Amide_ and MTR_Rex,NOE_ as a function of the actual B_1_ values in WM. The traces were obtained by segmenting the relative B_1_ map into the different regions between 50% and 150% in the steps of 1% and calculating the corresponding MTR_Rex,Amide_ and MTR_Rex,NOE_ contrast resulting from all available CEST datasets. The straight black lines represent the linear regression relation between the corresponding metrics and B_1_ in the B_1_ range 0.1–0.5 μT. The Pearson's correlation coefficient (R) and the corresponding *p*‐value are provided. ** represents statistical significance at the level *p* < 0.005.
**Sup. Figure S3.** The experimentally derived plots of the multiple Lorentzian fitting method quantified MTR_Rex,Amide_ and MTR_Rex,NOE_ as a function of the actual B_1_ values in WM. The traces were obtained by segmenting the relative B_1_ map into the different regions between 50% and 150% in the steps of 1% and calculating the corresponding MTR_Rex,Amide_ and MTR_Rex,NOE_ contrast resulting from all available CEST datasets. The straight black lines represent the linear regression relation between the corresponding metrics and B_1_ in the B_1_ range 0.1–0.5 μT. The Pearson's correlation coefficient (R) and the corresponding *p*‐value are provided. ** represents statistical significance at the level *p* < 0.005.
