# Supplementary material for: On the transmit field inhomogeneity correction of relaxation‐compensated amide and NOE CEST effects at 7 T
Source: NMR Biomed. 2017 Jan 23;30(5):e3687. doi: 10.1002/nbm.3687 (PMC5412922; doi:10.1002/nbm.3687)
Supplement: Supplementary file 1 — Sup. Figure S1. The four‐pool Bloch‐McConnell equation simulated B1 dependence of the effect size of (a) MTRRex,Amide and (b) MTRRex,NOE at various water T1 relaxation times. Sup. Figure S2. The experimentally derived plots of the three‐point method quantified MTRRex,Amide and MTRRex,NOE as a function of the actual B1 values in WM. The traces were obtained by segmenting the relative B1 map into the different regions between 50% and 150% in the steps of 1% and calculating the corresponding MTRRex,Amide and MTRRex,NOE contrast resulting from all available CEST datasets. The straight black lines represent the linear regression relation between the corresponding metrics and B1 in the B1 range 0.1–0.5 μT. The Pearson's correlation coefficient (R) and the corresponding p‐value are provided. ** represents statistical significance at the level p < 0.005. Sup. Figure S3. The experimentally derived plots of the multiple Lorentzian fitting method quantified MTRRex,Amide and MTRRex,NOE as a function of the actual B1 values in WM. The traces were obtained by segmenting the relative B1 map into the different regions between 50% and 150% in the steps of 1% and calculating the corresponding MTRRex,Amide and MTRRex,NOE contrast resulting from all available CEST datasets. The straight black lines represent the linear regression relation between the corresponding metrics and B1 in the B1 range 0.1–0.5 μT. The Pearson's correlation coefficient (R) and the corresponding p‐value are provided. ** represents statistical significance at the level p < 0.005. [file NBM-30-na-s001.doc]

**Supporting material**

**Effect of water T_1_ on B_1_-dependence of Amide and NOE effects**

**SI1**. The simulated data bellow shows the effect of water T_1_ on B_1_ dependence of the effect size of (**a**) MTR_Rex,Amide_ and (**b**) MTR_Rex,NOE_. While both Amide-CEST and NOE are scaled by water T_1_, the linear B_1_ dependence of these effects in the low B_1_ regime (0.15 - 0.50 µT) does not change with water T_1_. Both MTR_Rex,Amide_ and MTR_Rex,NOE_ effects were quantified by the pool difference method using the inverse metrics ([[1]](#endnote-2),[[2]](#endnote-3)) (**eq. 2** in the manuscript).

**

**

**Sup. Fig. S1**. The four-pool Bloch-McConnell equation simulated B_1_ dependence of the effect size of (**a**) MTR_Rex,Amide_ and (**b**) MTR_Rex,NOE_ at various water T_1_ relaxation times.

**Applicability of linear B_1_ correction to the three-point method**

**SI2**. Amide-CEST effect size was quantified by the three-point method ([[3]](#endnote-4)) using the inverse metrics (Error: Reference source not found^,Error: Reference source not found^):

(**Sup. eq. 1**)

where *MTR_Rex,Amide_* is the effect size of the cytosolic amides, *M_z_(Δω)* is the signal in the Z-spectrum at *Δω*, M_0_ is the equilibrium magnetization at the normalization offset Δ*ω = *500 ppm.

NOE effect size was quantified in a similar way by the three-point method using the inverse metrics (Error: Reference source not found^,Error: Reference source not found^):

(**Sup. eq. 2**)

where *MTR_Rex,NOE_* is the effect size of the cytosolic amides, *M_z_(Δω)* is the signal in the Z-spectrum at *Δω*, M_0_ is the equilibrium magnetization at the normalization offset Δ*ω = *500 ppm.


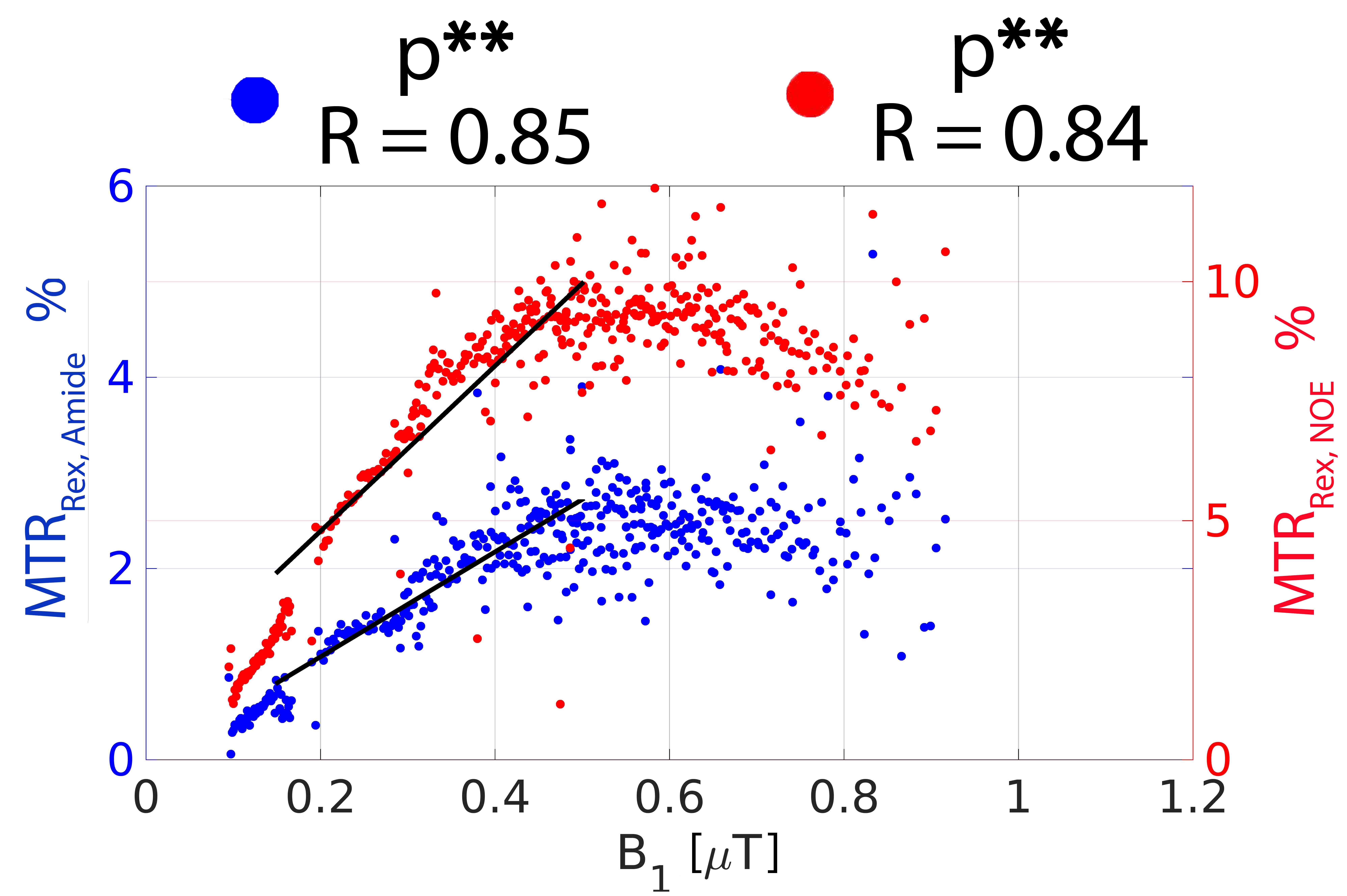


**Sup. Fig. S2.** The experimentally derived plots of the three-point method quantified MTR_Rex,Amide_ and MTR_Rex,NOE_ as a function of the actual B_1_ values in WM. The traces were obtained by segmenting the relative B_1_ map into the different regions between 50% and 150% in the steps of 1% and calculating the corresponding MTR_Rex,Amide_ and MTR_Rex,NOE_ contrast resulting from all available CEST datasets. The straight black lines represent the linear regression relation between the corresponding metrics and B_1_ in the B_1_ range 0.1 - 0.5 µT. The Pearson's correlation coefficient (R) and the corresponding p-value are provided. ** represents statistical significance at the level p<<0.005.

**Applicability of linear B_1_ correction to the multiple Lorentzian fitting method**

**SI3**. Amide CEST effect was quantified by the pool difference method (based on the six-pool multi-Lorentzian fitting method) ([[4]](#endnote-5)) using the inverse metrics (Error: Reference source not found^,Error: Reference source not found^):

(**Sup. eq. 3**)

where *MTR_Rex,Amide_* is the effect size of the cytosolic amides, *M_z_(Δω,M_b_)* is the signal in the Z-spectrum at *Δω *(*Δω* = 3.5 ppm for Amide-CEST), M_0_ is the equilibrium magnetization at the normalization offset Δ*ω = *500 ppm and *M_b_* is the amplitude of the Amide compartment (*M_b_* = 0 and *M_b_* = 1 for the system without and with Amide-CEST pool, respectively). A similar equation applies to the NOE pool (MTR_Rex,NOE_) at *Δω* = -3.5 ppm.


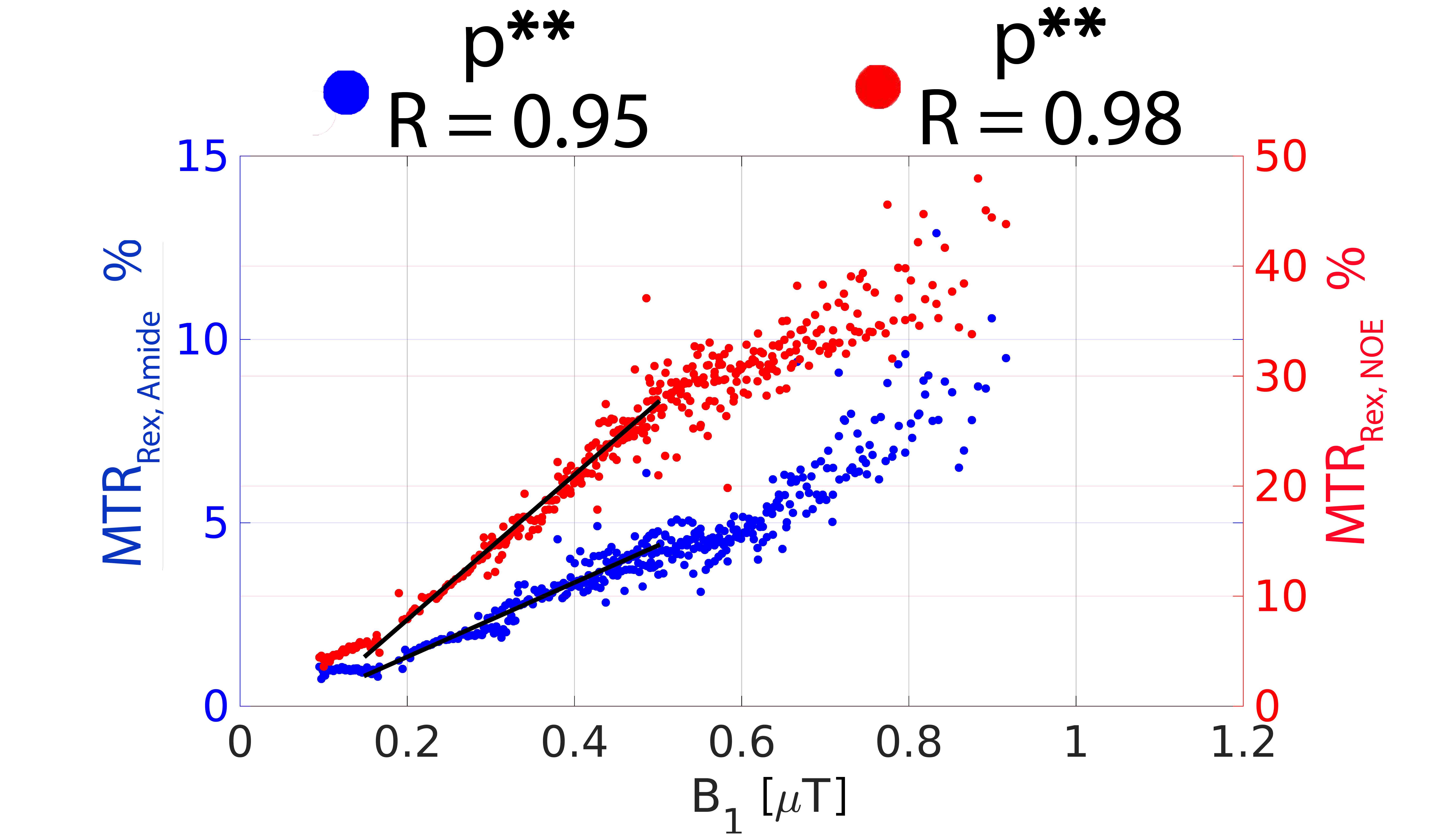


**Sup. Fig. S3.** The experimentally derived plots of the multiple Lorentzian fitting method quantified MTR_Rex,Amide_ and MTR_Rex,NOE_ as a function of the actual B_1_ values in WM. The traces were obtained by segmenting the relative B_1_ map into the different regions between 50% and 150% in the steps of 1% and calculating the corresponding MTR_Rex,Amide_ and MTR_Rex,NOE_ contrast resulting from all available CEST datasets. The straight black lines represent the linear regression relation between the corresponding metrics and B_1_ in the B_1_ range 0.1 - 0.5 µT. The Pearson's correlation coefficient (R) and the corresponding p-value are provided. ** represents statistical significance at the level p<<0.005.

**References**

1. Zaiss M, Xu J, Goerke S, Khan IS, Singer RJ, Gore JC, Gochberg DF, Bachert P. Inverse Z-spectrum analysis for spillover-, MT-, and T1-corrected steady-state pulsed CEST-MRI – application to pH-weighted MRI of acute stroke. NMR Biomed. 2014;27(3):240–252. [↑](#endnote-ref-2)
2. Zaiss M, Windschuh J, Paech D et al. Relaxation-compensated CEST-MRI of the human brain at 7T: Unbiased insight into NOE and amide signal changes in human glioblastoma. NeuroImage. 2015;112:180–188. [↑](#endnote-ref-3)
3. Jin T, Wang P, Zong X, Kim SG. MR imaging of the amide-proton transfer effect and the pH-insensitive nuclear overhauser effect at 9.4 T. Magn Reson Med. 2013;69(3):760–770. [↑](#endnote-ref-4)
4. Desmond KL, Moosvi F, Stanisz GJ. Mapping of amide, amine, and aliphatic peaks in the CEST spectra of murine xenografts at 7 T. Magn. Reson. Med. 2014; 71: 1841–1853. [↑](#endnote-ref-5)
